# Cardiovascular responses to leg-press exercises during head-down tilt

**DOI:** 10.3389/fspor.2024.1396391

**Published:** 2024-08-27

**Authors:** Cristiano Alessandro, Amirehsan Sarabadani Tafreshi, Robert Riener

**Affiliations:** ^1^ School of Medicine and Surgery, Sport and Exercise Medicine, University of Milano-Bicocca, Milan, Italy; ^2^ Sensory-Motor Systems Lab, Department of Health Sciences and Technology, Institute of Robotics and Intelligent Systems, ETH Zurich, Zurich, Switzerland; ^3^ Spinal Cord Injury Center, Medical Faculty, University of Zurich, Zurich, Switzerland

**Keywords:** gravitational load, orthostatic stress, exercise power, contraction frequency, blood pressure

## Abstract

**Introduction:**

Physical exercise and gravitational load affect the activity of the cardiovascular system. How these factors interact with one another is still poorly understood. Here we investigate how the cardiovascular system responds to leg-press exercise during head-down tilt, a posture that reduces orthostatic stress, limits gravitational pooling, and increases central blood volume.

**Methods:**

Seventeen healthy participants performed leg-press exercise during head-down tilt at different combinations of resistive force, contraction frequency, and exercise duration (30 and 60 s), leading to different exercise power. Systolic (sBP), diastolic (dBP), mean arterial pressure (MAP), pulse pressure (PP) and heart rate (HR) were measured continuously. Cardiovascular responses were evaluated by comparing the values of these signals during exercise recovery to baseline. Mixed models were used to evaluate the effect of exercise power and of individual exercise parameter on the cardiovascular responses.

**Results:**

Immediately after the exercise, we observed a clear undershoot in sBP (Δ = −7.78 ± 1.19 mmHg), dBP (Δ = −10.37 ± 0.84 mmHg), and MAP (Δ = −8.85 ± 0.85 mmHg), an overshoot in PP (Δ = 7.93 ± 1.13 mmHg), and elevated values of HR (Δ = 33.5 ± 0.94 bpm) compared to baseline (*p* < 0.0001). However, all parameters returned to similar baseline values 2 min following the exercise (*p* > 0.05). The responses of dBP, MAP and HR were significantly modulated by exercise power (correlation coefficients: r_dBP _= −0.34, r_MAP _= −0.25, r_HR _= 0.52, *p* < 0.001). All signals’ responses were modulated by contraction frequency (*p* < 0.05), increasing the undershoot in sBP (Δ = −1.87 ± 0.98 mmHg), dBP (Δ = −4.85 ± 1.01 and Δ = −3.45 ± 0.98 mmHg for low and high resistive force respectively) and MAP (Δ = −3.31 ± 0.75 mmHg), and increasing the overshoot in PP (Δ = 2.57 ± 1.06 mmHg) as well as the value of HR (Δ = 16.8 ± 2.04 and Δ = 10.8 ± 2.01 bpm for low and high resistive force respectively). Resistive force affected only dBP (Δ = −4.96 ± 1.41 mmHg, *p* < 0.0001), MAP (Δ = −2.97 ± 1.07 mmHg, *p* < 0.05) and HR (Δ = 6.81 ± 2.81 bpm, *p* < 0.0001; Δ = 15.72 ± 2.86 bpm, *p* < 0.0001; Δ = 15.72 ± 2.86 bpm, *p* < 0.05, depending on the values of resistive force and contraction frequency), and exercise duration affected only HR (Δ = 9.64 ± 2.01 bpm, *p* < 0.0001).

**Conclusion:**

Leg exercises caused only immediate cardiovascular responses, potentially due to facilitated venous return by the head-down tilt position. The modulation of dBP, MAP and HR responses by exercise power and that of all signals by contraction frequency may help optimizing exercise prescription in conditions of limited orthostatic stress.

## 1 Introduction

The cardiovascular system regulates hemodynamics under a variety of conditions, responding to stressors like physical exercise and gravity. Physical exercise challenges the cardiovascular system by requiring the delivery of an increased amount of oxygen to the muscles involved in the exercise, while maintaining appropriate levels of hemodynamics in the rest of the body (1). Gravity challenges the cardiovascular system by causing orthostatic stress, thereby reducing venous return and blood flow towards the upper body (2, 3). Whether challenged by exercise or by gravitational pooling, the cardiovascular system regulates several mechanisms to ensure appropriate blood flow and tissue oxygenation, for example by altering cardiac output and vasoconstriction (1, 2). This leads to observable changes in heart rate, stroke volume and blood pressure.

Physical exercise can be performed under a range of conditions that vary the amount of orthostatic stress and therefore the amount of gravitational pooling. First, exercises can be performed in an upright position, under orthostatic stress. In this situation, a reduction of arterial blood pressure compared to rest levels (i.e., post-exercise hypotension) is observed following both aerobic (4) and resistance exercises (5), along with a gradual decrease of heart rate after the elevation occurring during exercise. Moreover, following exercise there is a reduced stroke volume that, in the case of resistance training, contributes to a reduction of cardiac output (6). These cardiovascular responses are affected by post-exercise recovery posture. For example, the reduction of stroke volume is more pronounced during seated than during supine recovery following upright aerobic exercise (7–9), but not following resistance exercise (10). Similarly, in a seated recovery posture heart rate returns to rest level more slowly than in a supine position following both upright aerobic and resistance exercise (7). Finally, a more pronounced post-exercise hypotension is observed in seated than in supine recovery (7). In addition to recovery posture, cardiovascular responses to upright exercise are affected by exercise parameters. For example, the amount of post-exercise hypotension and, more generally, post-exercise hemodynamics are modulated by exercise intensity (11). While this parameter is typically quantified by the resistive force in resistance training, recent results suggest that also movement velocity, which determines muscle contraction frequency in repetitive movements, significantly affects exercise intensity (12) and post-exercise hemodynamics (13). Furthermore, cardiovascular responses are also affected by exercise volume (14). This parameter is typically quantified by the total number repetitions multiplied by the number of sets and resistive force (15, 16); therefore, it is directly related to the duration of each exercise set at a given value of resistive force and contraction frequency. In orthostatic conditions physical exercise therefore causes specific hemodynamic responses, modulated by recovery posture and several exercise parameters such as resistive force, contraction frequency and duration of each exercise set.

Exercise can also be performed with limited or no orthostatic stress. One extreme condition is exercise performed by astronauts during space flight [see e.g., (17, 18)], where individuals are no longer subjected to orthostatic stress and gravitational pooling. More commonly, similar conditions occur during supine or inclined exercise positions (19, 20) (e.g., while performing the leg-press), which alter the distribution of blood compared to an upright posture (21–23), hence stimulating the baroreceptors and affecting the mechanisms that regulate hemodynamics (2, 24). However, the cardiovascular responses to exercise performed under limited orthostatic stress, as well as how exercise parameters affect these responses, are still unclear. Filling this gap may contribute to a better understanding of the physiological processes of recovery from physical exercise (25) and may help optimizing exercise prescriptions in conditions of limited orthostatic stress (26).

**Figure 1:**
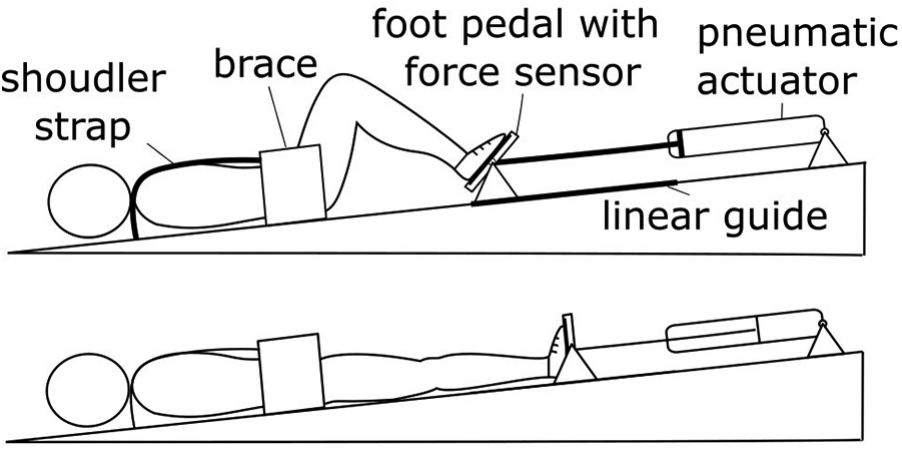
Experimental setup. The robotic device MARCOS was used to perform leg-press exercises during HDT. This system provides the user with a resistive force against leg-extension by means of pneumatic actuators, maintaining the desired force level during movement using fast feedback loops (additional details in the Method section).

In this study we evaluate the cardiovascular responses to leg-press exercises during head-down tilt (HDT). In this posture, subjects lie supine on a head-down tilted platform, leading to a displacement of body fluids towards the upper body. HDT is extensively used in space physiology (27), as it elicits similar cardiovascular responses to those observed during an extreme condition such as that of microgravity (28, 29). Therefore, HDT is an ideal setting to evaluate how the cardiovascular system responds to physical exercise with limited orthostatic stress. To this end, we developed a head-down tilted platform equipped with a robotic leg-press (Figure 1), that allowed us for the first time to perform exercise in this posture. Meanwhile, systolic, diastolic, mean arterial pressure, pulse pressure, and heart rate were continuously recorded to monitor the status of the cardiovascular system. Cardiovascular responses were assessed for different exercise parameters, evaluating how post-exercise hemodynamics are affected by resistive force, contraction frequency, and exercise duration in a condition of limited orthostatic stress.

## 2 Materials and methods

### 2.1 Participants

Seventeen male volunteers (see Table 1) with no history of cardiovascular nor musculoskeletal diseases signed an informed consent and participated in this study. All procedures were conducted according to the Declaration of Helsinki and were approved by the Ethical Review Committee of Canton Zurich.

**Table 1:** Participants anthropometrics and vital signs.

Participant (id)	Age (year)	Weight (kg)	Height (m)	BMI (kg/m^2^)	sBP (mmHg)	dBP (mmHg)	MAP (mmHg)	PP (mmHg)	HR (bpm)
1	34	93	1.80	28.7					
2	26	79.5	1.80	24.5	116	75	89	40	59
3	38	70.6	1.80	21.8	118	73	88	45	73
4	30	72.1	1.83	21.5	113	78	90	34	87
5	30	75.9	1.84	22.4	96	68	78	28	92
6	31	78.2	1.86	22.6	103	67	79	36	96
7	30	82.7	1.88	23.4	120	82	95	38	80
8	30	74.4	1.78	23.5	96	70	79	26	107
9	33	83.3	1.84	24.6	125	72	90	53	77
10	34	86	1.87	24.6	127	85	99	42	78
11	26	90	1.78	28.4	113	77	89	36	76
12	28	84.6	1.75	27.6	110	76	88	34	90
13	33	75	1.62	28.6	109	75	87	34	79
14	29	66	1.80	20.4	103	69	80	34	71
15	25	77	1.70	26.6	125	87	100	37	97
16	24	89	1.79	27.8	131	87	102	43	92
17	24	69.4	1.75	22.7	115	76	89	39	80
Mean ± S.D.	29.7 ± 3.9	79.2 ± 7.7	1.79 ± 0.06	24.7 ± 2.7	113.7 ± 10.5	76.2 ± 6.5	88.7 ± 7.4	37.5 ± 6.6	83.4 ± 11.9

The details of participants who took part in this study are reported. Vital signs were computed as the mean of each continuous cardiovascular signal across the last 90 s seconds of 3 min of recording in orthostatic position. Missing values in participant 1 were due to technical issues during the acquisitions.

### 2.2 Study protocol

Study participants were invited to the laboratory only once for this study. After signing the informed consent, two finger cuffs were secured to their index and medium fingers (right arm) in order to record continuous blood pressure (BP) measurements throughout the study (CNAP ® Monitor 500; CNSystem Medizintechnik AG, Austria); systolic (sBP), diastolic (dBP), mean (MAP), pulse pressure (PP) and heart rate (HR) were computed from this continuous BP signal as detailed in Section 2.5. An adjustable strap was used to support the arm of the participant in a standardized position, allowing the hand with the finger cuffs to rest at the level of the heart. Participants were initially asked to stand still in an upright position for 3 min to measure their vital signs. They were then invited to lie supine, legs extended, on a 6° head-down tilted platform equipped with a robotic leg-press device (see below, MARCOS). They were secured to the platform by means of straps and braces, and when they felt comfortable in this posture (1–2 min of acclimatation), participants were allowed to familiarize themselves with the leg-press by performing leg flexion-extension movements. Then, they were asked to keep their leg-extended against no resistance to start the experiment. Experiments started 5–10 min after the participant was secured to the tilted platform.

Participants performed nine consecutive experimental sessions, each consisting of three phases: baseline, exercise, and recovery. During baseline, participants kept their legs extended for 3 min against no resistance. In preparation for the exercise phase, they flexed their legs until reaching the mechanical stop of the leg-press (20 s). During the exercise phase, they performed leg-press exercises against the resistance of the device (either 30 or 60 s, see Exercise Protocol). Finally, during recovery, participants kept their legs extended for 5 min against no resistance. Experimental sessions were characterized by different exercise conditions (see Exercise Protocol). The order of these nine sessions was randomized across participants. In between sessions, the exercises phases were inherently separated by 8 min of rest, consisting of 5 min of washout of the current session plus 3 min of baseline of the following one. Participants remained on the head-down tilted platform for approximately 1.5 h to complete the full experiment. They were then helped to stand up and were allowed to sit down for as much time as they needed to feel comfortable before leaving the lab.

### 2.3 MARCOS

MARCOS is a robotic device originally developed to execute leg movements inside an MRI scanner, described in detail elsewhere (30). In this study, we adapted this device to perform leg-press exercises on a 6° head-down tilted platform (Figure 1). The load is realized by means of pneumatic actuators that can provide up to 400 N of resistance against leg-extension, comprising hip (range of motion: 0°−40°, with zero being the neutral position; extremes at the device mechanical stops) and knee flexion-extension (0°−70°, with zero being full extension), and ankle dorsi-plantar flexion movements (45°−90°, with 90° being the neutral ankle position). The user is secured to the platforms by means of: (1) shoes that are firmly attached to the foot pedals, (2) a brace that prevents mediolateral movements of the hip, and (3) shoulder straps that prevent sliding in the direction of force. The position of the brace as well as the length of the shoulder straps can be adjusted to the size of the user, allowing us to obtain very similar ranges of motion across participants.

### 2.4 Exercise protocol

Exercise condition was defined as a combination of the following parameters: (1) resistive force of the leg-press (two levels), F; (2) contraction frequency, f (0.5 or 1 Hz); (3) duration of the exercise phase, T (30 or 60 s). The two levels of resistive force were parametrized based on the weight of each participant: $F_{1i}=g\times \frac{w_{i}}{6}$ and $F_{2i}=g\times \frac{w_{i}}{3}$, where *w_i_* is the weight of participant *i* and *g *= 9.6 m/s^2^ is the gravitational acceleration. The frequency of the movement was controlled by asking the participants to follow a metronome, where each click corresponded to either leg-extension or leg-flexion, so that the period between consecutive leg-extensions was either 2 s or 1 s (obtaining the frequencies of 0.5 or 1 Hz, as stated above). We therefore tested eight conditions: all combinations of force, frequency, and duration levels. An additional experimental session was performed, in which the robotic leg-press applied resistance also during baseline and recovery; that session was not used for the present study.

### 2.5 Data acquisition and processing

From the BP signal we computed continuous traces of systolic BP (sBP), diastolic BP (dBP), heart rate (HR), mean arterial pressure (MAP) and pulse pressure (PP) as described previously (31). Briefly, sBP and dBP traces were obtained by interpolating the maximum and minimum peaks of the low-pass filtered BP traces (10 Hz, 3rd order Butterworth). Heart rate (HR) was estimated by the rate of adjacent systolic peaks. Finally, MAP was computed as 1/3 sBP + 2/3 dBP, and PP as the difference between the filtered systolic and diastolic BP traces.

We computed signal features at the baseline and recovery phases. We did not evaluate signals during exercise because the technique used by the CNAP monitor to measure continuous BP [i.e., vascular unloading technique using plethysmographic signals from infrared light sensors (32)] is affected by motion artefacts. During baseline the signals were stable; we therefore characterized their values by computing time averages over the last 90 s of this phase. During recovery, we evaluated an early response (early recovery: first 90 s of the recovery phase) and a late response (late recovery: last 90 s of recovery) to the leg-press exercise. During early recovery, there were rapid changes in the hemodynamics, that were quantified using signal-specific features (31, 33): Minimum of sBP, dBP and MAP (sBPmin, dBPmin, MAPmin); Maximum of PP (PPmax); Maximum of HR (HRmax). During late recovery (i.e., 3.5 min after the end of the exercise) the signals were stable, and therefore were characterized by their time averages.

### 2.6 Statistical analysis

To analyze the cardiovascular responses to leg-press exercise, we used Linear Mixed Effect Models (LMEM) using the nlme package in the R environment (34). LMEMs are statistical instruments that can cope with missing data (none in this study) and can take into account variability at different levels (across participants, across experimental phases, across experimental conditions). After fitting the LMEMs, we checked the assumptions of independence of residuals and random effects (35) by visually inspecting the distributions using qq-plots and histograms (these assumptions were always met in this study). Then, we performed analysis of variance (ANOVA) on the fitted models. If there were statistically significant differences in these general tests, we performed *post-hoc* comparisons to evaluate statistical differences between experimental conditions on the dependent variable under investigation, using two-tail *z*-tests and adjusting the *p*-values using Bonferroni corrections. We considered differences to be statistically significant if the *p*-value for the null-hypothesis was <0.05.

Initially, we considered the time evolution of each signal across the experimental session, evaluating differences between baseline (before the leg-press exercise) and recovery phase (after the exercise). We fit a LMEM to the features of each signal across these phases; i.e., time average at baseline, signal-specific features at early recovery, time average at late recovery. We considered *phase* (baseline, early recovery, late recovery) as the fixed-effect (independent variable), and *subject-id* as a random-effect, effectively implementing a repeated-measure analysis (measurements of each signal at different time points, for each participant) on a one-way ANOVA with factor *phase*. In this analysis we pooled all experimental conditions, hence obtaining multiple observations for each phase and participant (all 8 combinations of force, frequency, and duration levels). This analysis allowed us to evaluate statistically significant changes of the signal values across the experimental phases independently on exercise parameters.

We then evaluated whether the cardiovascular responses to leg-press exercises were modulated by exercise power. First, we estimated the power associated to each exercise condition (i.e., each combination of force, frequency and duration levels) for each participant as *F *× *d *× *f*, where *F* is the resistive force applied by the leg-press, *d* is the distance traveled by the foot pedals during leg extension (measured using the encoders mounted on MARCOS) and *f* is the frequency of the leg-extension movements. Since exercise duration does not influence power calculation, we obtained two measurements (associated with the two exercise durations) for each power within each participant. Then the correlations between these measures and post-exercise responses were analyzed. To this end, we initially checked for outliers in the values of each signal-specific feature relative to baseline (ΔsBPmin, ΔdBPmin, ΔMAPmin, ΔPPmax, ΔHRmax), across experimental conditions and participants. Outliers were defined as datapoints more than 1.5 inter-quartile ranges above or below the first or the third quartile respectively (Tukey's method). After removing the detected outliers, we computed the Pearson correlation between exercise power and the remaining signal features for each signal.

To analyze the effects of individual exercise parameters on post-exercise responses, we fit a LMEM to the values of each signal-specific feature at early recovery with respect to baseline. Before fitting, these features were transformed with square root (ΔsBPmin, ΔdBPmin, ΔMAPmin, ΔPPmax) or logarithm (ΔHRmax) to render their distributions approximately normal. Similarly, we tested the effect of individual exercise parameters on the average force and on the average peak force applied to each foot pedal (i.e., left and right leg) throughout the exercise phase by fitting a LMEM to each of these variables, for the left and the right legs separately. For both analyses, we considered *resistive force* (high and low), *contraction frequency* (0.5 Hz, 1 Hz), *exercise duration* (30 s, 60 s) and their interactions as fixed-effects (independent variables), and *subject-id* as a random-effect (effectively implementing a three-ways ANOVA). *Post-hoc* tests were then conducted to compare the values of the feature across the levels of each exercise parameter.

## 3 Results

### 3.1 Cardiovascular responses to leg-press exercises

**Figure 2:**
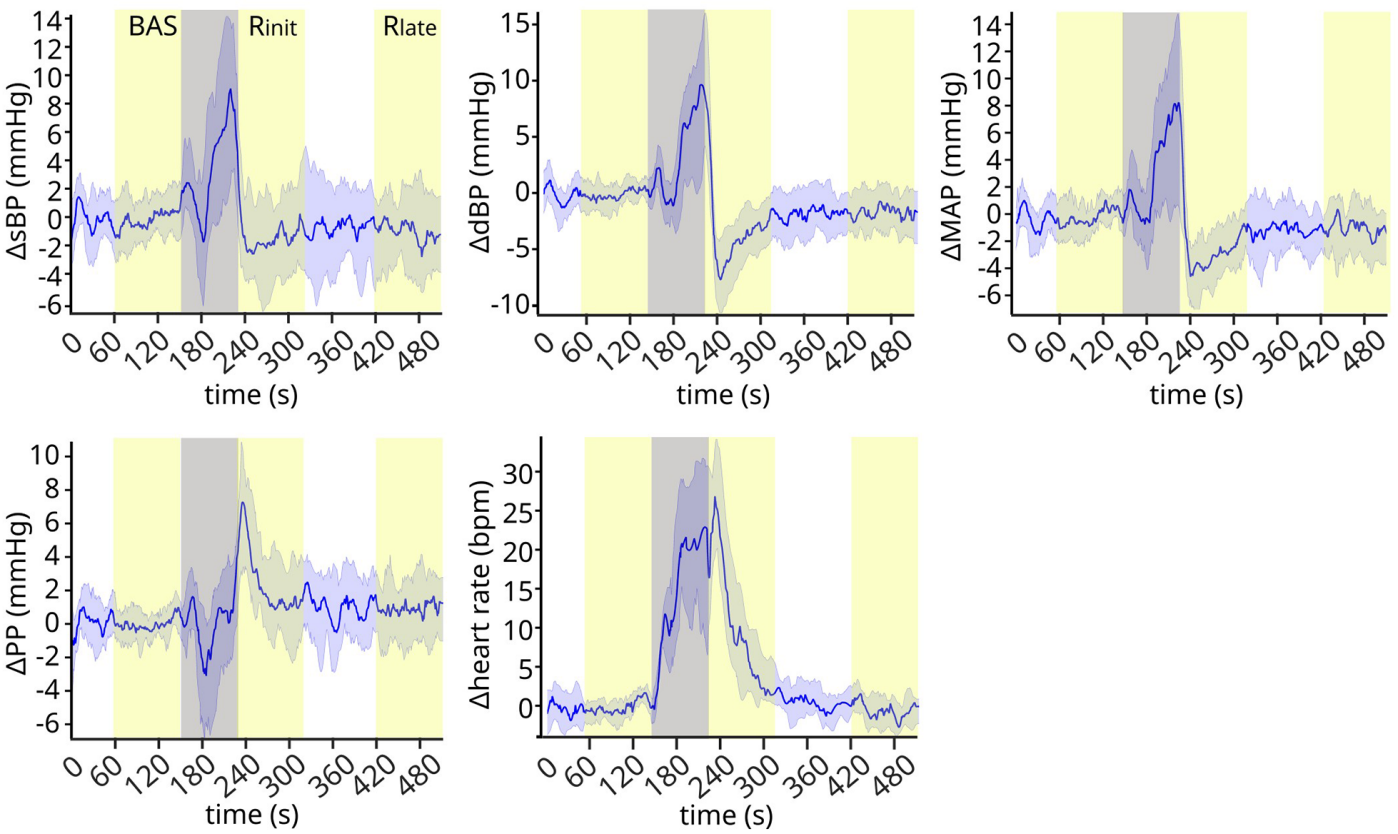
Cardiovascular signals throughout the experiment. The cardiovascular signals exhibit clear trends after the exercise phase, showing well-defined transient dynamics during early recovery and stabilizing at later time points. Gray band: preparation and exercise phase. Yellow bands: baseline (BAS), early recovery (R_init_), and late recovery (R_late_). Signals are normalized by subtracting the baseline mean values for each subject, and then represented in the plot as mean ± standard deviation (s.d.) across subjects. *N* = 17.

The cardiovascular signals follow clear trends throughout the experimental phases (Figure 2). While they are stable during baseline, they exhibit well-defined variations shortly after the exercise phase (early recovery), and then stabilize again around 2 min after the exercise phase (late recovery). Systolic, diastolic and mean blood pressure decrease rapidly reaching a low peak (undershoot) during early recovery, and then trend towards higher values at late recovery. On the contrary, pulse pressure shows a high spike at early recovery, before stabilizing to lower values during late recovery. Finally, heart rate decreases monotonically during early recovery and stabilizes during late recovery.

**Figure 3:**
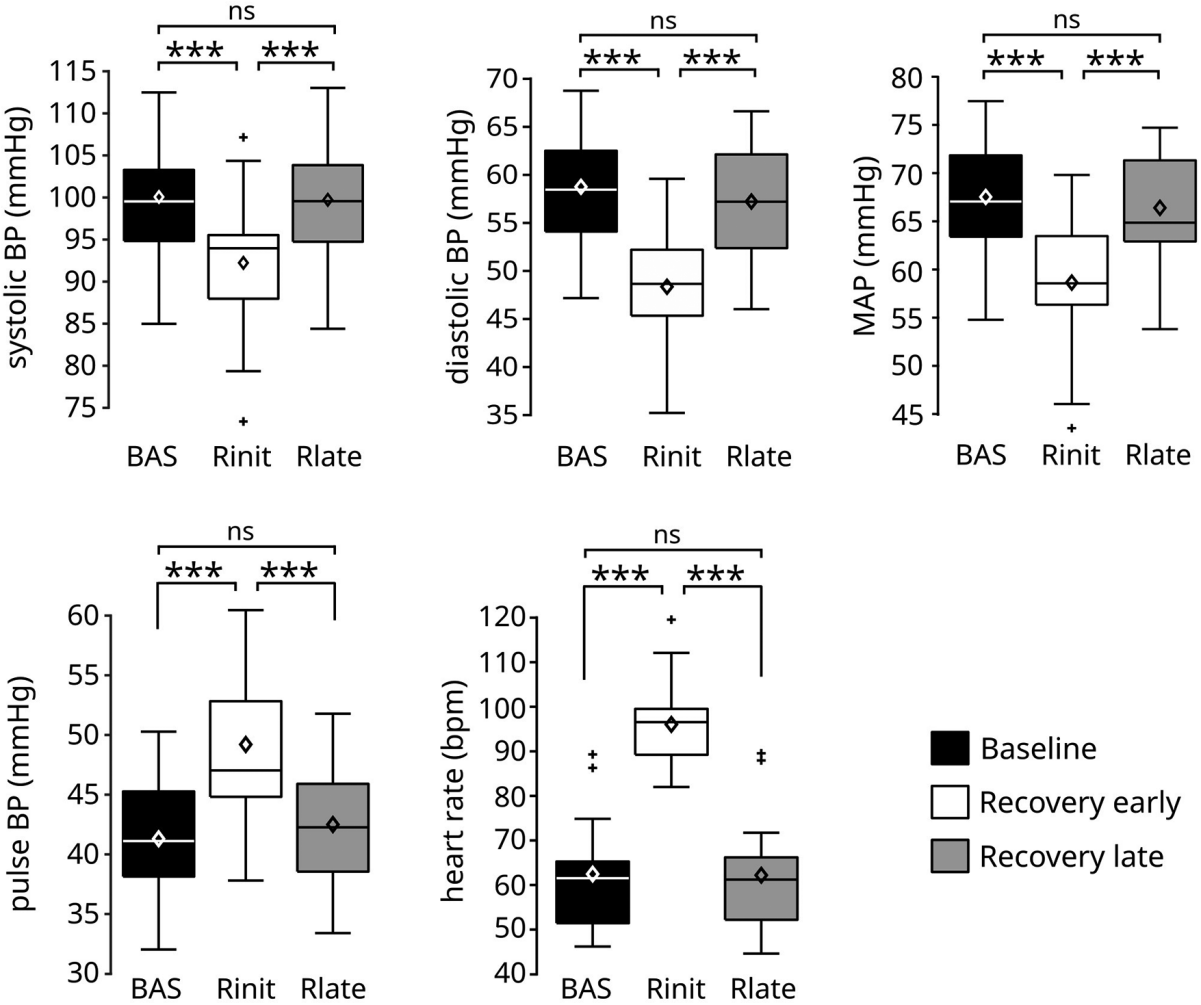
Values of the cardiovascular signals before and after the exercise phase. The execution of the leg-press exercise resulted in a significant reduction of systolic, diastolic, and mean arterial pressure, and in a significant increase of pulse blood pressure and heart rate during early recovery (R_init_) compared to baseline (BAS). All signals returned to similar baseline values during late recovery (R_late_). Data are presented as box and whiskers plots, indicating mean values with diamonds. *N* = 17. ***p < 0.0001.

These qualitative observations were confirmed from the statistical analysis of the signals across experimental phases (Figure 3). The ANOVA indicated a significant effect of experimental phase for all signals (sBP *p* < 0.0001; dBP: *p* < 0.0001; MAP: *p* < 0.0001; PP: *p* < 0.0001; HR: *p* < 0.0001). *Post-hoc* tests showed that the early recovery values were significantly different from those observed at both baseline and late recovery for all signals (Table 2): the values in sBP (Δ = −7.78 ± 1.19 mmHg), dBP (Δ = −10.37 ± 0.84 mmHg) and MAP (Δ = −8.85 ± 0.85 mmHg) were significantly lower, and the overshoot in PP (Δ = 7.93 ± 1.13 mmHg) as well as the maximum value in HR (Δ = 33.5 ± 0.94 bpm) were significantly higher than the corresponding baseline and late recovery values. All signals then returned to similar baseline values later in the recovery phase, so that no statistically significant differences were found between baseline and late recovery for any signal.

**Table 2:** *Post-hoc* tests to compare the values of the cardiovascular signals across experimental phases.

	sBP		dBP		MAP		PP		HR	
Comparison	*p*	Δ (mmHg)	*p*	Δ (mmHg)	*p*	Δ (mmHg)	*p*	Δ (mmHg)	*p*	Δ (bpm)
Rinit-BAS	**<0** **.** **0001**	−7.78 ± 1.19	**<0**.**0001**	−10.37 ± 0.84	**<0**.**0001**	−8.85 ± 0.85	**<0**.**0001**	7.93 ± 1.13	**<0**.**0001**	33.5 ± 0.94
Rlate-Rinit	**<0**.**0001**	7.47 ± 1.19	**<0**.**0001**	8.85 ± 0.84	**<0**.**0001**	7.73 ± 0.85	**<0**.**0001**	−6.72 ± 1.13	**<0**.**0001**	−33.8 ± 0.95
Rlate-BAS	1.000		0.207		0.555		0.847		1.000	

Comparisons are indicated as differences between the cardiovascular responses observed in two experimental phases. For example, Rinit-BAS indicates the difference between the cardiovascular responses obtained at early recovery and baseline. *P*-values are reported for each comparison; mean differences (Δ) are reported only if the corresponding test is significant. Significant *p*-values are highlighted in bold.

### 3.2 Cardiovascular responses as a function of exercise power

**Figure 4:**
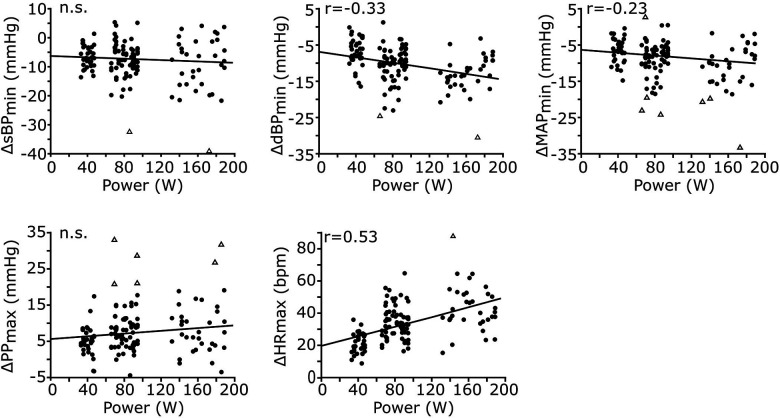
Correlations between exercise power and cardiovascular responses during early recovery. The cardiovascular responses of all signals across participants and exercise conditions are reported against exercise power (*n* = 137 data points for each plot, resulting from 17 participants and 8 conditions). All responses, except those for sBP and PP, are significantly correlated with exercise power. While the responses in dBP and MAP are negatively correlated, those in HR are positively correlated with exercise power. The Pearson correlation coefficient (*r*) is reported only for significant correlations; otherwise, non-significance (n.s.) is reported.

Since the execution of leg-press exercise at HDT caused significant cardiovascular responses during early recovery, we evaluated whether those responses were modulated by exercise power (Figure 4). Exercise power was not correlated to the undershoot in sBP (*p* = 0.27) or to the overshoot in PP (*p* = 0.08). However, it was weakly negatively correlated with the undershoot in dBP (correlation coefficient *r* = −0.33, *p* < 0.001) and MAP (*r* = −0.23, *p* = 0.009), and moderately positively correlated with the maximum value of HR (*r* = 0.53, *p* < 0.001) observed at early recovery.

### 3.3 Cardiovascular responses as a function of individual exercise parameters

#### 3.3.1 Influence of individual exercise parameters on produced forces

**Figure 5:**
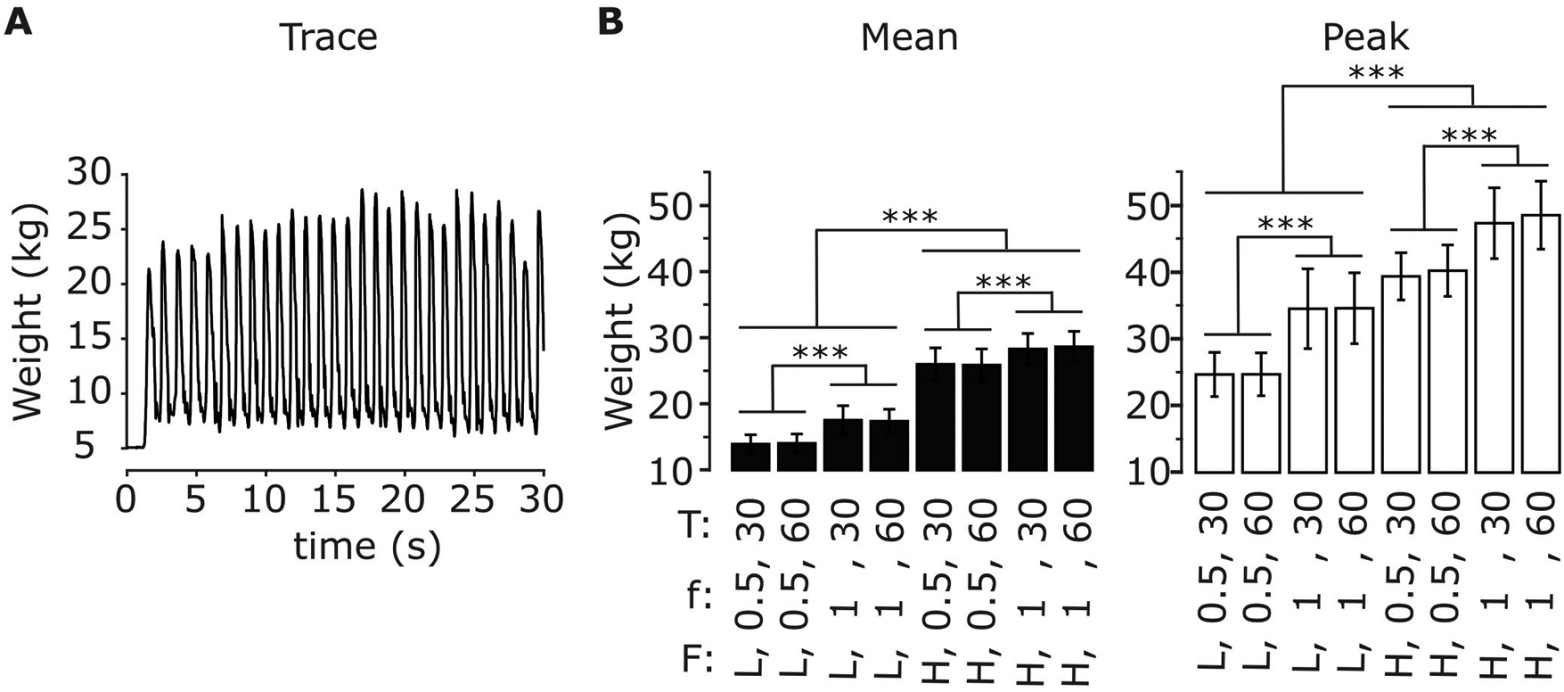
Applied forces during exercise for different exercise parameters. **(A)** Time-varying force applied to the left foot pedal for a representative subject during exercise (low force, *f* = 1 Hz, *T* = 30 s). **(B)** Mean values of the time-varying force applied to the left foot pedal (left) and mean value of the signal high peaks (right) across the exercise phase for all exercise parameters (resistive force F: low (L), high (H); contraction frequency f: 0.5, 1 Hz; exercise duration T: 30, 60 s). Data are represented as mean ± s.d. across subjects (*N* = 17). *** *p* < 0.0001.

Figure 5A shows the time-varying force applied by a representative participant to the left foot pedal during an exercise session (low force, *f* = 1 Hz, *T* = 30 s). The force values exhibit oscillations associated to each movement repetition, with greater values during leg extension (as the device opposes movement) and lower values during leg flexion (as the device facilitates movement). We evaluated how the mean value of the applied force throughout the exercise as well as the mean value of the high peaks of the applied force differed across exercise conditions (Figure 5B). The ANOVA indicated a significant effect of resistive force, contraction frequency and their interaction on the mean force value for both the left and the right leg (Table 3). *Post-hoc* tests then revealed that the mean force applied by the participants’ left leg increases with the resistive force of the leg-press for both contraction frequencies (*f* = 0.5 Hz: Δ = 11.9 ± 0.24 kg, *p* < 0.001; *f* = 1 Hz: Δ = 11.0 ± 0.24 kg, *p* < 0.001), as well as with contraction frequency for both values of resistive force (F_low_: Δ = 3.47 ± 0.24 kg, *p* < 0.001; F_high_: Δ = 2.54 ± 0.24 kg, *p* < 0.001). Similar results were obtained for the right leg, obtaining an increase in mean applied force with resistive force for any contraction frequency (*f* = 0.5 Hz: Δ = 11.8 ± 0.23 kg, *p* < 0.001; *f* = 1 Hz: Δ = 10.9 ± 0.23 kg, *p* < 0.001), and with contraction frequency for any resistive force (F_low_: Δ = 3.48 ± 0.23 kg, *p* < 0.001; F_high_: Δ = 2.58 ± 0.23 kg, *p* < 0.001). The mean applied force of the participants varies between 14 ± 0.48 kg (F_low_, *f* = 0.5 Hz) and 28.5 ± 0.48 Kg (F_high_, *f* = 1 Hz) in the left leg, and between 13.7 ± 0.47 kg (F_low_, *f* = 0.5 Hz) and 28.0 ± 0.47 Kg (F_high_, *f* = 1 Hz) in the right leg. Similarly, the ANOVA indicated a significant effect of resistive force and contraction frequency on the mean peak force values, with an interaction term between these two parameters just below significance for the left leg and significant for the right leg (Table 3). Peak force increased with resistive force (Δ = 14.2 ± 0.45 kg, *p* < 0.001) and contraction frequency (Δ = 8.99 ± 0.45 kg, *p* < 0.001) in the left leg. Similarly, it increased with resistive force for any contraction frequency (*f* = 0.5 Hz: Δ = 15.4 ± 0.59 kg, *p* < 0.001; *f* = 1 Hz: Δ = 13.4 ± 0.59 kg, *p* < 0.001), as well with contraction frequency for any resistive force (F_low_: Δ = 9.86 ± 0.59 kg, *p* < 0.001; F_high_: Δ = 7.94 ± 0.59 kg, *p* < 0.001) in the right leg. Mean peak force value varied between 29.6 ± 0.96 kg (F_low_) and 43.8 ± 0.96 Kg (F_high_) in the left leg, and between 23.7 ± 1.03 kg (F_low_, *f* = 0.5 Hz) and 47.0 ± 1.03 Kg (F_high_, *f* = 1 Hz) in the right leg.

**Table 3:** ANOVA tables to evaluate the effects of the exercise parameters on the applied force.

Exercise parameter	Mean L	Mean R	Peak L	Peak R
Force	**<0** **.** **001**	**<0**.**001**	**<0**.**001**	**<0**.**001**
Frequency	**<0**.**001**	**<0**.**001**	**<0**.**001**	**<0**.**001**
Duration	0.814	0.479	0.237	0.612
Force: frequency	**0**.**007**	**0**.**006**	0.058	**0**.**022**
Force: duration	0.688	0.633	0.283	0.541
Frequency: duration	0.787	0.642	0.827	0.572
Force: frequency: duration	0.271	0.468	0.872	0.580

The *p*-values associated with each exercise parameter (force, frequency, and duration) and their interactions are reported for the mean values (mean) and the mean peak values (peak) of the force applied by the left (L) and the right (R) legs. Significant *p*-values are highlighted in bold.

#### 3.3.2 Influence of individual exercise parameters on early cardiovascular responses

**Figure 6:**
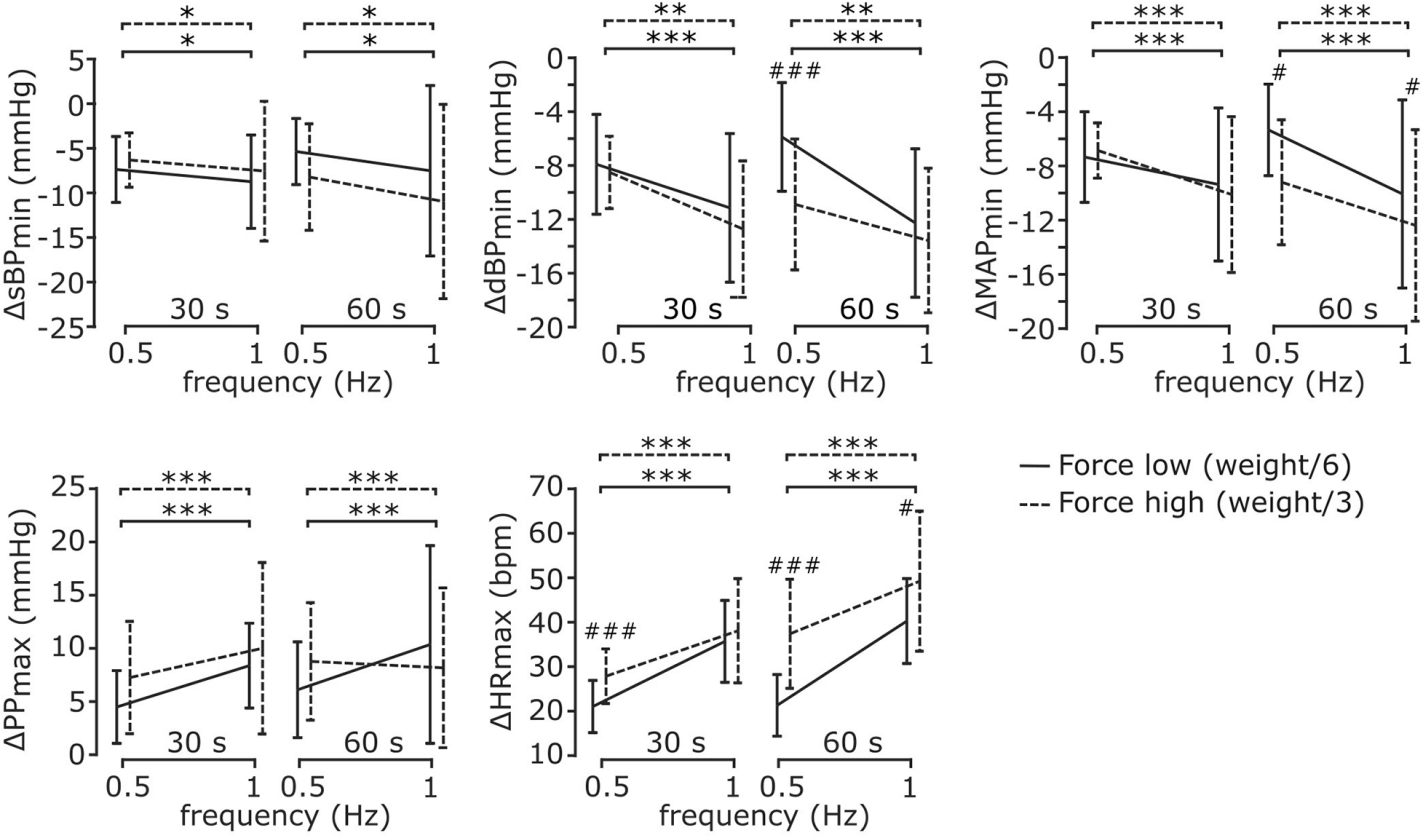
Cardiovascular responses during early recovery for different exercise parameters. All cardiovascular responses were significantly affected by contraction frequency, but only some of them were affected by resistive force and exercise duration. Details on the analysis are reported in the text. Data are represented as mean ± s.d. across subjects (*N* = 17). * Statistical difference between contraction frequencies; # statistical difference across resistive forces; * # *p* < 0.05, ** ## *p* < 0.001, *** ### *p* < 0.0001.

Figure 6 illustrates how early cardiovascular responses are affected by each exercise parameter; the corresponding statistical results reported in Table 4. The ANOVA indicated that early response in sBP was only significantly affected by contraction frequency, so that its values were greatly diminished following a fast contraction frequency of 1 Hz compared to a slower one of 0.5 Hz (*post-hoc*). The responses in dBP were significantly affected by resistive force and contraction frequency, with a significant interaction between resistive force and exercise duration (ANOVA). In particular, the early-response reduction in dBP was greater following a fast (1 Hz) compared to a slower (0.5 Hz) contraction frequency. Furthermore, such a reduction was significantly more pronounced following the exercise against the high resistive force compared to that against the low resistive force, although this difference only appeared for an exercise duration of 60 s at 0.5 Hz contraction frequency (*post-hoc*). The ANOVA of early MAP responses indicated a significant effect of contraction frequency, and a significant interaction between resistive force and exercise duration. According to *post-hoc* tests, early response MAP values were greatly reduced following a fast (1 Hz) compared to a slow (0.5 Hz) contraction frequency, and following a 60 s exercise against the high compared to the low resistive force. Early PP responses were only significantly affected by contraction frequency (ANOVA), so that their values greatly increased following exercises at high contraction frequency (*post-hoc*). Finally, the ANOVA of early responses in HR indicated significant effects for all exercise parameters, and significant interactions between resistive force and contraction frequency as well as between resistive force and exercise duration. In particular, HR responses increased with contraction frequency for all resistive forces, as well as with resistive force at both 0.5 Hz (for both exercise durations) and 1 Hz contraction frequency (only following a 30 s duration). Finally, HR responses increased with the duration of exercise, but only when they were performed against the high resistive force.

**Table 4:** ANOVA tables and *post-hoc* tests to compare the cardiovascular responses during early recovery across exercise parameters.

ANOVA table	ΔsBP_min_		ΔdBP_min_		ΔMAP_min_		ΔPP_max_		ΔHR_max_	
Force	0.113		**0**.**002**		0.098		0.114		**<0**.**001**	
Frequency	**0**.**011**		**<0**.**001**		**<0**.**001**		**0**.**001**		**<0**.**001**	
Duration	0.273		0.821		0.570		0.454		**<0**.**001**	
Force: frequency	0.866		0.064		0.176		0.308		**<0**.**001**	
Force: duration	0.105		**0**.**047**		**0**.**011**		0.795		**0**.**014**	
Frequency: duration	0.673		0.493		0.397		0.711		0.348	
Force: frequency: duration	0.340		0.110		0.422		0.294		0.322	
*Post-hoc* comparison	*p*	*Δ* (mmHg)	*p*	*Δ* (mmHg)	*p*	Δ (mmHg)	*p*	Δ (mmHg)	*p*	Δ (bpm)
Δfrequency (F_low_, T_30_)	**0**.**007**	−1.87 ± 0.98	**<0**.**0001**	−4.85 ± 1.01	**<0**.**0001**	−3.31 ± 0.75	**<0.0001**	2.57 ± 1.06	**<0**.**0001**	16.8 ± 2.04
Δfrequency (F_low_, T_60_)										
Δfrequency (F_high_, T_30_)			**0**.**004**	−3.45 ± 0.98					**<0**.**0001**	10.8 ± 2.01
Δfrequency (F_high_, T_60_)										
Δforce (f_0.5_, T_30_)			0.525		1.000				**<0**.**0001**	6.81 ± 2.81
Δforce (f_0.5_, T_60_)			**<0**.**0001**	−4.96 ± 1.41	**0**.**011**	−2.97 ± 1.07			**<0**.**0001**	15.72 ± 2.86
Δforce (f_1_, T_30_)			1.000		1.000				1.000	
Δforce (f_1_, T_60_)			0.396		**0**.**011**	−2.97 ± 1.07			**0**.**021**	8.27 ± 2.91
Δduration (F_low_, f_0.5_)			1.000		0.767				1.000	
Δduration (F_low_, f_1_)										
Δduration (F_high_, f_0.5_)			0.769		0.090				**<0**.**0001**	9.64 ± 2.01
Δduration (F_high_, f_1_)										

The ANOVA *p*-values associated with each exercise parameter (force, frequency, and duration) and their interactions are reported for each cardiovascular response. *Post-hoc* test comparisons are indicated as differences between the cardiovascular responses obtained for the two levels of the parameter under investigation, for a given value of the other two parameters. For example, Δfrequency (F_low_, T_30_) indicates the comparison of cardiovascular responses for the two levels of repetition frequency (response a 1 Hz minus response at 0.5 Hz), given a low force level and an exercise duration of 30 s. Differences are always computed between the high and the low value of the parameter under investigation. *P*-values are reported for each comparison (significant in bold); mean differences (Δ) are reported only if the corresponding test is significant. *Post-hoc p*-values and mean differences are reported for groups of tests when the ANOVA indicates that only some of the exercise parameters significantly affect the cardiovascular responses. As an example, a single *p*-value and mean difference is reported for all rows that evaluate the effect of frequency (Δfrequency) on the response of systolic blood pressure (ΔsBP_min_). Indeed, the ANOVA (see Table 2) indicates that repetition frequency is the only exercise parameter that affects sBP response, without interaction with other parameters; therefore, the effect of repetition frequency is independent of the values of other parameters, leading to a single *p*-value and mean differences.

## 4 Discussion

### 4.1 Summary of results

We investigated the cardiovascular responses to leg-press exercises during HDT, a posture that eliminates orthostatic stress hence limiting gravitational pooling (29). To this end, we employed a tilted platform embedded with a robotic leg-press that maintains a controlled level of resistive force during leg extension. This method allowed us to evaluate the cardiovascular responses to exercises at different conditions, defined by combinations of resistive force, repetition frequency and exercise duration. The execution of the exercise caused significant after-effects on the cardiovascular signals during early recovery: an undershoot in systolic, diastolic, and mean arterial pressure, an overshoot in pulse pressure and a monotonic decrease in heart rate. However, these after-effects vanished 2 min after the end of the exercise phase, when all the signals reached baseline values. The magnitude of the after-effects at early recovery was correlated to exercise power for all signals but systolic and pulse pressure, and was mainly associated with contraction frequency. On the contrary, resistive force and exercise duration were only partially associated to the magnitude of cardiovascular responses, affecting mainly heart rate and blood pressure depending on the levels of other exercise parameters.

### 4.2 Physiology of recovery from exercise

The recovery period from exercise elicits clear physiological responses, distinct from those observed during rest or physical activity (25). While the physiology of such a recovery state is still not fully understood (36), it likely involves local vasodilation (37) and resetting of the baroreflex characteristics leading to a reduction in heart rate and sympathetic outflow (38, 39). In addition, the loss of muscle pump from contracting muscles may reduce venous return (40). In orthostatic conditions, these alterations lead to the phenomenon of post-exercise hypotension, which may last for hours after the end of the exercise bouts (41) and may cause syncope if not properly regulated by homeostatic mechanisms (40).

The condition of HDT used in this study led to different cardiovascular responses from those expected in orthostatic condition. Consistent with the phenomenon of post-exercise hypotension, we observed a transient decrease in blood pressure during early recovery. However, this effect only lasted for less than 5 min; i.e., the values of blood pressure observed during late recovery were not significantly different from baseline. We therefore did not observe persistent post-exercise hypotension, similarly to (42) following prolonged aerobic exercise and to (10) following resistance exercise. This effect may not have appeared for at least three reasons. First, HDT substantially limited venous pooling, hence facilitating venous return despite a potential post-exercise vasodilation in the lower limbs and the loss of muscle pump. This effect may also explain the transient increase in pulse blood pressure observed in this study (Figures 2, 3) (43). Consistently, Tarso et al. (10) showed an absence of post-exercise hypotension following resistance exercise during supine (but not seated) recovery, a posture that like HDT limits orthostatic stress. Second, the exercise was not intense enough to elicit persistent vasodilation. While we did not quantify 1 repetition maximum (RM) in this study (see Limitations below), we showed that performing the exercise at 1 Hz against the high resistive load led to peaks of 47.0 ± 1.03 Kg (left leg, similar values for the right; see Section 3.3.1), which is approximately 33% RM in recreational athletes (44, 45). On the other hand, Duncan at al. observed post-exercise hypotension at 80% RM but not at 40% RM following resistance exercise under orthostatic stress (46). Whether those results also apply in the absence of orthostatic stress is unclear. Further, the fitness level of our participants was not reported here, rendering it difficult to compare their% RM with those of recreation athletes. Nonetheless, these considerations certainly suggest that additional experiments need to be performed to better evaluate the effect of exercise intensity on persistent vasodilation in our experimental conditions. If exercise intensity was not sufficient to elicit persistent vasodilation, the observed transient decrease in blood pressure during early recovery may have been caused by immediate postexercise hyperemia (47). Third, post-exercise hypotension would be observed later than the duration of the recovery phase considered here. While previous research has indeed shown that the lowest blood pressure values are observed a few hours after training, a milder drop in blood pressure should already appear a few minutes after the end of an exercise bout (11). We therefore believe that we did not observe post-exercise hypotension because of the absence of orthostatic stress due to the HDT posture, although additional experiments on longer and more intense exercise sessions should be performed to confirm this hypothesis.

We observed that following exercise, heart rate decreased monotonically and returned to baseline values in less than 5 min. This monotonic decrease is qualitatively expected, and originate from the concurrent re-activation of parasympathetic activity (which was depressed during exercise) and withdrawal of sympathetic activity (which was hyperactive during exercise) during recovery (48). However, quantitatively, previous studies showed a persistent increase in heart rate 30–60 min following both resistance (10) and aerobic (39) training in orthostatic condition. This difference may partially originate from the increase in parasympathetic activity during HDT (49, 50). Consistently, previous studies showed a faster heart rate recovery in supine than in seated position following both aerobic (8) and resistance exercise (10) (executed in upright position). Yet, in some of these studies (7) [but not all (10)] heart rate remained elevated compared to baseline 10–20 min following exercise, potentially due to the execution of different exercise at a higher intensity than those included in this study.

Previously, we showed that the HDT protocol used here elicited a reduction of BP and HR and no change in PP compared to upright position at rest (31). These responses likely result from baroreflex activity evoked by the increase in central blood volume, that reduces peripheral vasoconstriction and HR, leading to diminished BP values (51). Similar responses are observed here early after the execution of the exercise. The transient reduction of BP early after exercise, along with the concurrent increase in PP, are likely due to increased vasodilation following a post-exercise resetting of the baroreflex characteristics (25), and to reduced vessel compression following muscle relaxation (52) [consistently, BP undershoot reduces if continuous muscle loading occurs following exercise (31)]. It is possible that the magnitude of the post-exercise baroreflex resetting during HDT is higher than in orthostatic conditions, since HDT itself already causes a baroreflex resetting towards lower BP and HR values (53). Potentially, this may lead to a more pronounced post-exercise BP undershoot during HDT than in orthostatic conditions. However, these hypotheses will need to be verified in future studies.

### 4.3 Effect of exercise power and exercise parameters on cardiovascular responses to physical exercise

Previous research showed that during the execution of physical exercise there is a power-dependent shift in the characteristics of the baroreflex (1), allowing appropriate modulation of heart rate and blood pressure based on exercise demands. Whether exercise power influences cardiovascular responses during recovery is still controversial, with inconsistent results across studies (41). Furthermore, most of this previous research evaluated this phenomenon hours after the exercise sessions in orthostatic condition, making it difficult to make comparison with the present experiments that evaluate short-term effects during HDT.

In this study we showed that during HDT, some of the cardiovascular responses (dBP, MAP and HR) to physical exercise observed at early recovery were modulated by exercise power (Figure 4). The physiological mechanisms underlying this result are unclear. It is possible that the shift of the baroreflex characteristic associated to the increased central blood volume (24, 54, 55) (due to the HDT posture) interacts with the shift caused by the execution of the exercise (1, 56), potentially affecting the restoration of such characteristics during recovery in a power-dependent manner. Another possibility is that the power-dependent responses observed here emerge as after-effects of the muscle pump mechanism caused by the execution of the leg-press; i.e., increasing exercise power results in increased venous return (57), which in turn may cause an increase in the undershoot in diastolic and mean arterial pressure, and an increase in the overshoot in pulse blood observed during early recovery (Figure 4). This idea is consistent with the fact that the magnitude of the cardiovascular responses observed here was mainly associated with contraction frequency (Figure 6), a parameter highly related to the effects of the muscle pump (58, 59). Finally, while PP responses did not appear to be modulated by exercise power, the Pearson correlation between these variables was relatively close to significance (*p* = 0.08). Since the outliers’ detection method used here can be conservative (60), we cannot rule out that PP is in fact modulated by exercise power. Additional experiments need to be performed to test these ideas and re-evaluate this aspect.

We showed that exercise parameters influence both the applied force on the foot pedals (Figure 5) and the early cardiovascular responses following exercise (Figure 6). In particular, the former was affected by contraction frequency and (as expected) resistive force, while the latter was influenced mainly by contraction frequency and not always by resistive force. This observation confirms that resistive force [e.g., with respect to RM (61)] is not the sole determinant of the cardiovascular responses following exercise, and that contraction frequency should be clearly considered when evaluating the effects of training on the cardiovascular system. Such a result is in line with recent studies that indicate movement velocity as a major determinant of exercise intensity (12). While those studies quantified this idea in terms of movement performance, here we show that contraction frequency affects all cardiovascular responses under investigation early after exercise. Potentially, these results may originate from the muscle pump mechanism, which is clearly affected by contraction frequency (9, 58, 59). In addition, they could also result from the intensity-based modulation of the baroreflex resetting and of the muscle metaboreflex during exercise (1), hence affecting heart rate and blood pressure responses early after exercise. In terms of exercise volume, our results showed that exercise duration has a limited effect on post-exercise blood pressure [consistent with that found in (62) during aerobic exercise) but affects early heart rate responses (Section 3.3.2). The increase of heart rate with exercise duration may be related to cardiovascular drift (63) and by a delay in parasympathetic re-activation following longer exercise (64).

### 4.4 Implications on training under limited orthostatic stress

While the current study was not aimed at developing an optimized training regime in conditions of limited orthostatic stress, the obtained results may provide useful information in that context. For example, we showed that contraction frequency is a major determinant of the transient decrease of BP following leg-press exercise during HDT. Therefore, if the aim of the training was to decrease blood pressure without excessively loading the heart, training may consist in short bouts of movement repetitions performed at a high contraction rate against a low resistive force; the latter, along with a relatively low duration of each exercise bout should limit the increase in HR (see Section 3.3.2, Figure 6). Vice versa, if the aim was to maximize the load while minimizing BP responses, training may consist of bouts of low-frequency contractions against a heavy resistive force.

While these considerations may apply to physical exercise performed in supine or inclined positions during standard gym sessions, they may also have implications in the context of space physiology. Daily physical training is indeed prescribed to astronauts during space missions as a countermeasure to ameliorate the negative effects of microgravity on the cardiovascular, the musculoskeletal and the sensorimotor systems (65, 66). Microgravity causes an unloading of the vestibular system that leads to a reduction of movement accuracy [likely due to untuned internal models (67)], and a shift of body fluid to the upper body that leads to both short- and long-term effects on the cardiovascular system; these include a reduction of blood pressure and heart rate, as well as a reduction of baroreflex sensitivity that ultimately leads to orthostatic intolerance upon returning to earth gravity condition (68, 69). The introduction of a daily training routine ameliorates these effects eliciting long-term effects on the cardiovascular system (17).

Some of the short-term cardiovascular responses observed in our current and previous work following the execution of leg-press exercises may potentially contribute to reducing cardiovascular deconditioning. For example, the observed increase in heart rate and in leg-blood volume (33) contrast the decreasing trends of these signals due to microgravity (68, 69). In addition, we recently showed that continuous leg-muscle loading also re-establishes earth-like values of blood pressure (31). This motivates the development of wearable devices that can provide astronauts with such a continuous leg-muscle loading, and that allow them to perform short bouts of leg-press-like exercises. Recent developments of soft exoskeletons (70) may indeed be used for such a purpose, providing the user with appropriate trunk muscle loading and resistive force against leg extension movements. These ideas will need to be tested in future studies. To this end, the effect of intermittent short bouts of leg-press exercises will be assessed during HDT stimulations of longer duration (i.e., days instead of minutes), hence eliciting a similar cardiovascular adaptation to that observed in microgravity (71, 72).

### 4.5 Limitations

The main limitation of this work is that we did not evaluate cardiovascular responses to exercises in orthostatic condition. These additional experiments would have allowed us to perform a direct comparison to the results presented here, isolating the effect of gravitational loading on exercise-induced cardiovascular responses. However, a fair comparison would have required us to perform identical exercises in both conditions (i.e., same movement kinematics and exercise parameters), a very hard requirement to satisfy. For example, squat exercises and horizontal leg-press involve different movement kinematics and kinetics (73). Even if we used a tilt table with adjustable inclination angle to maintain movement kinematics, in orthostatic conditions the resistive force to leg extension would be affected by body weight (20), hence influencing muscle activation (74, 75). Alternatively, one could impose a static muscle loading equal to body weight during the resting phases in HDT (31) (i.e., limited orthostatic stress), and then repeat the exercise on the same machine after tilting the table in a head-up setting (i.e., orthostatic stress). Differently from our current setting, this would require MARCOS to be mounted on a tilt table. Since the cardiovascular responses to physical exercise in orthostatic conditions are fairly documented already (1, 25, 76–80), we decided to focus on the effect of physical exercise during HDT.

In this study, the resistive force of the device was modulated based on body weight, rather than on percentages of maximum voluntary contraction (MVC) or RM. Those normalization methods are typically used to define similar relative resistive forces across potentially heterogeneous participants. However, given that none of the participants in this study were professional athletes and that they were normal weighted (average BMI of 24.8 kg/m^2^), body weight provides an indication of muscle strength (81, 82). Such a measure has recently been indicated as the anthropometric scaling parameter that reduces inter-subject variability in muscle strength (83), an issue that significantly affects normalization methods based on MVC (84). Nonetheless, other methods to directly ensure similar relative resistive force across participants should be used in future studies to validate the current results.

While we believe that 8 min of rest in between the exercise phases of sequential session (i.e., 5 min of washout plus 3 min of baseline) is enough to recover from 30 to 60 s of exercise, we cannot exclude that the sequential execution of the exercise sessions may have introduced a confounding effect of fatigue. Similarly, due to the absence of a warm-up phase in our protocol, we cannot exclude a gradual adaptation of the cardiovascular responses throughout the experiment. These confounding factors have been tackled by randomizing the exercise sessions across participants. It is therefore very unlikely, if not impossible, that these effects introduced consistent trends in the present results. Rather, they may have increased the variability in the dataset, which further reinforces the reliability of our statistically significant results.

In the current study, the execution of the exercise session started a few minutes after placing the participant in the HDT platform. We have allowed enough time for the cardiovascular signals to stabilize before starting the first session (see Section 2.5), and we previously showed that this timing leads to significant cardiovascular adaptations to the reduced orthostatic stress (31). Nonetheless, a longer period in the HDT posture is needed to elicit comparable cardiovascular adaptations to those obtained following long-term exposure to microgravity (27–29). While this was out of the scope of the current study, it will be interesting to evaluate these aspects in future studies. In that context, it will be important to introduce direct measurements of cardiac output and stroke volume; indeed, using PP as a surrogate for stroke volume, like we did here, may underestimate the values of this variable (85). In addition, we will need to evaluate potentially differential effects between male and female participants (86–88). The absence of this comparison certainly represents a limitation of the current study, that will need to be addressed in future.

## 5 Conclusions

This work describes the dynamics of the cardiovascular signals following short bouts of leg exercises in the absence of orthostatic stress. It shows that exercise power modulates cardiovascular responses in dBP, MAP and HR during early recovery. Furthermore, it identifies contraction frequency as the exercise parameter that most affects these responses, leading to a decrease in sBP, dBP and MAP, and to an increase in PP and HR. Future research will address strategies to optimize training based on this parameter, and will evaluate the effects of such training long after the exercise session.

## Data Availability

The raw data supporting the conclusions of this article will be made available by the authors, without undue reservation.
